# Non‐Linear Dysanaptic Lung Growth in Patients With Post‐Infectious Bronchiolitis Obliterans

**DOI:** 10.1002/ppul.71716

**Published:** 2026-07-03

**Authors:** James M. Clegg, Ferdinand Cacho, Matthew D. McGraw, Michael G. O'Connor

**Affiliations:** ^1^ Department of Pediatrics, Division of Pediatric Pulmonology Vanderbilt University Medical Center Nashville Tennessee USA; ^2^ Department of Pediatrics, Division of Pediatric Pulmonology University of Rochester Medical Center Rochester New York USA

**Keywords:** adenovirus, *Mycoplasma pneumoniae*, PIBO, pulmonary function

## Abstract

**Introduction:**

Few studies have assessed longitudinal pulmonary function outcomes in post‐infectious bronchiolitis obliterans (PIBO). This study analyzes pulmonary function changes from patients with PIBO at a single center with respect to time and inciting respiratory pathogen.

**Methods:**

Patients with PIBO were identified via retrospective observation using the Research Derivative at Vanderbilt University Medical Center. Serial spirometry was analyzed by generalized mixed‐effects models. An exploratory analysis was performed to test interactions with pathogen, age at sentinel infection, and systemic corticosteroid exposure.

**Results:**

Fifteen patients were diagnosed with PIBO from 1998 to 2023. Twelve (80%) were male. Median age of infection was 3.0 years (IQR: 1.3–6.5 years). Commonly identified pathogens were adenovirus (*n* = 8/15) and *Mycoplasma pneumoniae* (*n* = 4/15). Patients with *M. pneumoniae* were older at infection (*p* = 0.02). Eleven (73%) patients performed a median of 14 pulmonary function tests per patient (IQR: 6.5−23). Median *z*‐scores at initial spirometry were forced expiratory volume in 1 second (FEV1) −3.83, forced vital capacity (FVC) −2.92, and FEV1/FVC ratio −2.61. FEV1, FVC, and FEV1/FVC *z*‐scores changed by 0.008, 0.185, and −0.143/year, respectively (*p* = 0.69, 0.003, 0.004). Four of 9 (44%) patients tested were bronchodilator responsive. The rate of annual FVC *z*‐scores improved more in adenovirus‐infected patients than in those with other pathogens (p < 0.001).

**Conclusion:**

Patients with PIBO demonstrate non‐linear dysanaptic lung growth with stagnant FEV1 growth with respect to time. Subjects infected with adenovirus demonstrated lower FVC initially but accelerated FVC growth than those infected with other pathogens. Future research is needed on how severe respiratory infections contribute to non‐linear dysanaptic lung growth in susceptible children.

## 1 Introduction

Bronchiolitis obliterans (BO) is a recognized diffuse lung disease of the small airways. BO is characterized histologically by concentric airway fibrosis that results in obliteration of the airway lumen. Definitive diagnosis requires a lung biopsy, which is considered the gold standard. However, most patients affected by BO are diagnosed using clinical criteria with known risk factors, chest imaging, and pulmonary function testing (PFT). Risk factors include need for hospitalization due to an inciting infection, invasive ventilatory support and intensive care, as well as the persistent need for supplemental oxygen following hospitalization. Computed tomography (CT) of the chest demonstrates mosaic attenuation, with areas of ground‐glass opacity and air trapping. With time, bronchiectasis develops due to airway obstruction and mucus impaction [1, 2, 3, 4].

In children, BO is most commonly seen following severe respiratory tract infection, termed “post‐infectious BO” or PIBO [5]. While many pathogens are associated with PIBO, adenovirus is the most common [1, 5, 6, 7, 8, 9]. Other respiratory pathogens affiliated with PIBO include influenza, respiratory syncytial virus (RSV), and *Mycoplasma pneumoniae* (*M. pneumoniae*) [5]. The mechanisms that contribute to PIBO development remain poorly understood and are thought to include an exaggerated inflammatory response following lower respiratory tract infection that then triggers peribronchiolar fibrosis of the non‐cartilaginous small airways [1, 4].

Spirometry in PIBO demonstrates an obstructive phenotype with decreased forced expiratory volume in 1 second (FEV1), decreased forced vital capacity (FVC), and a decreased FEV1/FVC ratio [1]. Despite a low FVC, body plethysmography demonstrates normal total lung capacity (TLC) and a significant increase in residual volume (RV), resulting in a high RV/TLC ratio, indicative of air trapping. This high RV/TLC ratio drives the decrease in FVC [1]. It has been widely accepted that obstruction associated with PIBO is typically not responsive to bronchodilator challenge [1]; however, recent reports have identified some patients that are bronchodilator‐responsive [10, 11, 12, 13].

Despite the impact of PIBO on lung function, there have been only a handful of case series on quantitative long‐term pulmonary function and lung growth patterns in children with PIBO [10, 14, 15, 16, 17, 18]. The aim of this study is to report on the longitudinal spirometry trends in patients with PIBO diagnosed in a single center, as well as exploratory analyses regarding inciting pathogen, steroid exposure, and age of infection within the set.

## 2 Materials and Methods

**Figure 1:**
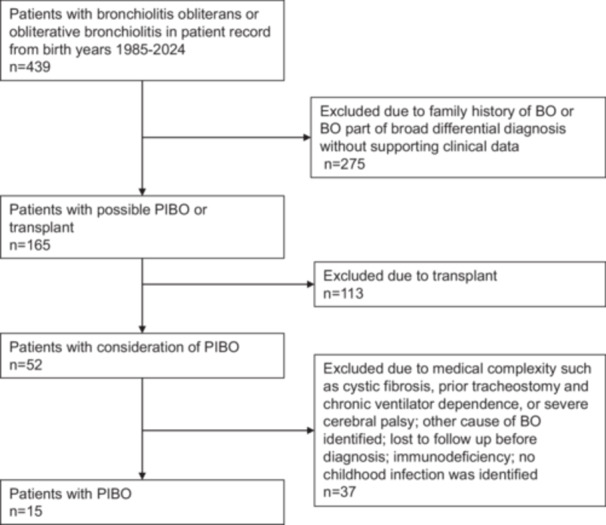
Flowchart for identification and study selection of patients with post‐infectious bronchiolitis obliterans. Using the Research Derivative at Vanderbilt University Medical Center, patient charts from birth years 1985 to 2024 were searched for “bronchiolitis obliterans” or “obliterative bronchiolitis” in their medical chart, as well as ICD9 and ICD10 codes for “Restrictive Bronchiolitis Interstitial Lung Disease.” Records were then screened to identify previously healthy patients who developed PIBO. Inclusion criteria were previously healthy patients who were diagnosed by a pediatric pulmonologist at Vanderbilt University Medical Center with PIBO after a severe lower respiratory tract infection, and exclusion criteria included significant prior respiratory or developmental history, such as prior tracheostomy or chronic ventilator dependence, primary ciliary dyskinesia, or cystic fibrosis.

The study was a retrospective observational study of previously healthy patients who were evaluated and diagnosed with PIBO by pediatric pulmonologists at Vanderbilt University Medical Center (VUMC). The VUMC internal review board reviewed the study protocol and granted exempt status because it posed minimal risk to participants. Informed consent was not required due to the retrospective nature of the study. Patients were identified using the Research Derivative (RD) at VUMC. The RD is a database designed for research, comprised of existing clinical data from VUMC electronic medical records [19, 20]. Any patient that had “bronchiolitis obliterans” or “obliterative bronchiolitis” mentioned in their chart, including inpatient notes, outpatient notes, radiology or pathology reports, family history, nursing reports, or problem list, was queried in the RD. ICD9 code 516.34 and ICD10 code J84.115—Respiratory Bronchiolitis Interstitial Lung Disease were also used in the query. Review of the resultant patient records identified previously healthy patients who were diagnosed with PIBO by a pediatric pulmonologist at VUMC (Figure 1).

### 2.1 Study Population

Medical records of patients born between 1985 and 2024 were screened for PIBO, limited to these years by relatively few electronic medical records for the first decade of life in patients born in the early years of this range. The date of hospitalization was used as the date of infection. Physician diagnosis of PIBO was made based on a history of severe pulmonary infection in otherwise healthy individuals, typical CT findings, and no other apparent cause for chronic severe pulmonary disease [1, 21]. The date of diagnosis was determined by the physician's documentation. Patients with significant prior respiratory or developmental history, such as prior tracheostomy or chronic ventilator dependence, primary ciliary dyskinesia, or cystic fibrosis, were excluded. Other exclusion criteria included a history of transplant, immunodeficiency, and other etiologies of BO being identified (Figure 1). Those with a prior diagnosis of asthma were not excluded.

### 2.2 PFT

PFT of spirometry and plethysmography was performed by qualified technicians according to protocols from the appropriate American Thoracic Society (ATS), or ATS/European Respiratory Society joint guidelines [22, 23, 24]. Measurements of FEV1 in liters, FVC in liters, TLC in liters, RV in liters, age, sex, and height from the PFT results were analyzed using the Global Lung Function Initiative race‐neutral calculator, ensuring all data points were evaluated equally [25]. Spirometry results were analyzed against time since infection for uniformity, as the age of infection varied greatly. Bronchodilator response was determined by ([post‐bronchodilator value [L] – pre‐bronchodilator value [L]]/predicted value) × 100, according to ERS/ATS lung function technical standards [26]. Available plethysmography data were reviewed for the impact of PIBO on RV and TLC.

In exploratory analyses with respect to age, 5 years of age at infection was used to divide patients, as this is an age that most patients can perform reliable PFT. This allowed for a time‐independent variable of pulmonary function.

### 2.3 Statistics

The Mann−Whitney *U* test determined the statistical significance of differences in age at infection between *M. pneumoniae* and other pathogens. Mixed effects analysis (two‐way ANOVA with Šídák's correction for multiple comparisons) determined differences in lung function of adenovirus versus non‐adenovirus infected patients with respect to group and time. Generalized mixed‐effects regression models evaluated changes in pulmonary function over time, with restricted maximum likelihood estimation applied for parameter estimation. Time since infection was a fixed effect, and random effects were specified at the individual level to account for repeated measures for each patient. To assess potential non‐linear relationships between time and pulmonary function, restricted cubic splines with four knots were incorporated into the model. The corresponding knot locations were approximately 0.15 (5%ile), 1.31 (35%ile), 3.83 (65%ile), and 8.95 years (95%ile). Further exploratory analyses compared interactions between time and infection (adenovirus vs. other, or *M. pneumoniae* vs. other), age of infection, or systemic corticosteroid exposure. Leave‐one‐out sensitivity analysis was performed to verify exploratory analysis findings. A *p* < 0.05 was assumed to represent statistical significance. Statistical analyses and figure generation were performed using R (version 4.5.1; R Core Team, 2025), GraphPad Prism (version 10.6.1; GraphPad Software, Boston, MA, USA; www.graphpad.com), and Stata (version 18.0; StataCorp LLC, College Station, TX, USA).

## 3 Results

### 3.1 Patient Characteristics

Fifteen previously healthy patients diagnosed with PIBO were identified via retrospective chart review from 1998 to 2023 (Table 1). Twelve of 15 (80%) patients were male. The median age at time of initial infection was 3.0 years (interquartile range [IQR]: 1.3−6.5 years). The median age of PIBO diagnosis was 4.8 years (IQR: 2.9−7.4 years). Eleven patients were born full term, one was born late preterm, and three patients had no gestational age noted in the record. All patients identified in this study required hospitalization at the time of initial infection, and all but one required supplemental oxygen during hospitalization. Pathogens identified by viral culture, nasal swab PCR, or serum titers included adenovirus in 8 (53%) patients and *M. pneumoniae* in 4 (27%). Two patients were co‐infected with adenovirus and *M. pneumoniae*. Four (27%) patients were co‐infected with either adenovirus or *M. pneumoniae* plus one or multiple other pathogens. One patient had RSV, and one had influenza that was complicated by methicillin‐resistant *Staphylococcus aureus* pneumonia and bacteremia. Three (20%) patients had unidentified pathogens. The median age at the time of *M. pneumoniae* infection was 7.5 years (IQR: 6.6−8.3 years), which differed significantly from other pathogens (*p* = 0.02). All patients had at least one feature (mosaic attenuation, air trapping, and/or ground glass) on chest CT commonly affiliated with PIBO [2, 3, 4]. Eleven (73%) patients developed bronchiectasis. Three (20%) patients underwent lung biopsy.

**Table 1:** Individual patient characteristics.

Patient	Age at infection (years)	Age at diagnosis (years)	Time to diagnosis (years)	Sex	Pathogen	Max respiratory support	CT findings	Biopsy features	PFT^a^
1	0.3	0.8	0.5	F	Unknown	LFNC	MA, AT		Yes
2	1.5	5.5	4	M	AdV, R/E	HFNC	MA, AT, B		Yes
3	2.8	4	1.2	F	Unknown	LFNC	AT, B		Yes
4	3	3.8	0.8	M	AdV	LFNC	GGO		Yes
5	4.7	4.8	0.1	M	AdV	HFNC	MA, AT, B		Yes
6	5.3	7.3	2	F	CMV, *Mp*	LFNC	MA		Yes
7	5.8	6.1	0.3	M	Unknown	None	MA, AT, GGO, B	Intraluminal foam cells, inflammatory bronchiolitis	Yes
8	7.1	7.4	0.3	M	AdV, *Mp*	LFNC	MA, B		Yes
9	8	8.1	0.1	M	AdV, *Mp*	HFNC	MA, AT, GGO, B		Yes
10	9.1	10	0.9	M	Flu, MRSA	NIPPV	MA, AT, GGO, B	Bronchiolar obliterative foci	Yes
11	9.3	9.5	0.2	M	*Mp*	NIPPV	MA, AT, GGO		Yes
12	0.6	1.6	1	M	AdV, HPIV‐3, HPIV‐4	Intubation	MA, B		No
13	0.7	0.8	0.1	M	RSV	Intubation	MA, AT, B		No
14	1.1	2	0.8	M	AdV, R/E, hMPV	HFNC	MA, AT, B		No
15	2.8	3.7	0.9	M	AdV	LFNC	MA, AT, B	Bronchiectasis, chronic bronchiolitis, and obliterative foci	No
	3.0 (1.3, 6.5)^b^	4.8 (2.9, 7.4)^b^	0.8 (0.2, 1.0)^b^						

Abbreviations: AdV, adenovirus; AT, air trapping; B, bronchiectasis; CMV, cytomegalovirus; CT, computed tomography; d, days; GGO, ground glass opacities; HFNC, high flow nasal cannula; hMPV, human metapneumovirus; HPIV‐3, human parainfluenza 3; HPIV‐4, human parainfluenza 4; LFNC, low flow nasal cannula; MA, mosaic attenuation; *Mp*, *Mycoplasma pneumoniae*; MRSA, methicillin‐resistant staphylococcus aureus; NIPPV, noninvasive positive pressure ventilation; PFT, pulmonary function test; R/E, rhinovirus/enterovirus; RSV, respiratory syncytial virus.

^a^ See Table 2 for more data.

^b^ Represents median (interquartile range) for column.

### 3.2 Post‐Infection Interventions

Five of 15 (33%) patients required home supplemental oxygen (Supporting Information S1: E‐Table 1). Five (33%) patients received long‐term oral corticosteroids, and 7 (47%) received pulse dose steroids. Thirteen (87%) patients received inhaled corticosteroids (ICS), while seven of these patients also received combination long‐acting beta agonist therapy. Nine (60%) patients were prescribed azithromycin, and 2 (13%) received montelukast. Two (13%) patients received intravenous immunoglobulin. One (7%) patient received etanercept and tiotropium as adjunctive therapies. Nine (60%) patients were prescribed airway clearance protocols. One patient required a lobectomy, but none of the patients died or progressed to lung transplant.

### 3.3 PFT

**Figure 2:**
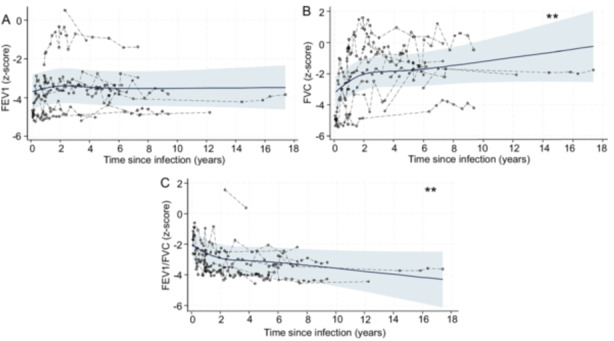
Relationship of spirometry and time since infection. Generalized mixed‐effects regression models were used to evaluate changes in pulmonary function over time, with restricted maximum likelihood estimation (REML) applied for parameter estimation. Population trendline (navy blue line), 95% confidence interval (shaded area), and individual pulmonary function outcomes (open circles with dashed lines) are represented. (A) Forced expiratory volume in 1 second (FEV1) *z*‐score versus time since infection. (B) Forced vital capacity (FVC) *z*‐score versus time since infection. (C) FEV1/FVC ratio *z*‐score versus time since infection. **p < 0.01. [Color figure can be viewed at wileyonlinelibrary.com]

Eleven of 15 (73%) patients performed spirometry (Table 2). Four patients did not perform spirometry due to age (*n* = 3) or severity of illness (*n* = 1). From 11 patients, 165 spirometry events were analyzed with a median of 14 events/patient (IQR: 6.5−23). The time from infection to initial spirometry was 0.8 years (IQR: 0.2, 2.8 years) at a chronological age of 6.3 years (IQR: 5.7, 7.6 years). Median *z*‐scores for FEV1, FVC, and FEV1/FVC at the time of initial spirometry were −3.83 (IQR: −4.72, −3.14), −2.92 (IQR: −4.57, −1.61), and −2.61 (IQR: −3.20, −2.08), respectively. The time from initial to final spirometry was 6.4 years (IQR: 3.2−8.6 years). Median *z*‐scores for FEV1, FVC, and FEV1/FVC at the time of final spirometry were −3.82 (IQR: −4.81, −3.16), −1.55 (IQR: −2.12, −1.21), and −3.62 (IQR: −4.30, −3.15). Over 6 years' duration, there was no significant change in FEV1 *z*‐score with respect to time (0.008/year; 95% confidence interval (CI): −0.029, 0.045; *p* = 0.688). Conversely, FVC *z*‐score increased non‐linearly with an average annual increase of 0.185/year (CI: 0.061, 0.309; *p* = 0.003), while the annual rate of decline for the FEV1/FVC *z*‐score was −0.143/year (CI: −0.239, −0.046; *p* = 0.004) (Figure 2; Table 3). Intraclass correlation coefficient was 0.955 (CI: 0.894, 0.981) for FEV1, 0.886 (CI: 0.752, 0.952) for FVC, and 0.934 (CI: 0.841, 0.974). Bronchodilator response was assessed in nine patients who performed spirometry. Four (*n* = 4/9; 44%) patients demonstrated a positive bronchodilator response. Eight (*n* = 8/11; 73%) patients performed plethysmography, demonstrating median *z*‐scores TLC 1.38 (IQR: 1.25, 1.92), RV 2.02 (IQR: 1.70, 3.93), and RV/TLC ratio 2.77 (IQR 2.17, 4.37, Supporting Information S2: E‐Figure 1).

**Table 2:** Pulmonary function outcomes for individual patients.

Patient^a^	Age at first PFT (years)^b^	Pathogen	Time from infection to first PFT (years)	Time difference first to final PFT (years)	First FEV1/FVC *z*‐score	Final FEV1/FVC *z*‐score	RV *z*‐score	TLC *z*‐score	RV/TLC *z*‐score	BDR
1	6.3	Unknown	6.0	11.4	2.75	3.62	1.83	0.08	2.50	No
2	5.3	AdV, R/E	3.8	2.2	3.28	3.33	*Not tested*	*Not tested*	*Not tested*	No
3	5	Unknown	2.2	1.5	0.10	1.23	1.71	1.28	2.26	*Not tested*
4	6.3	AdV	3.3	8.8	3.95	4.43	*Not tested*	*Not tested*	*Not tested*	Yes
5	5.1	AdV	0.4	1.7	3.11	4.33	*Not tested*	*Not tested*	*Not tested*	*Not tested*
6	6.2	CMV, *Mp*	0.9	6.4	2.44	2.19	1.44	2.14	1.60	Yes
7	6.1	Unknown	0.3	7.0	2.61	3.11	2.21	1.25	2.26	No
8	7.1	AdV, *Mp*	0	5.6	1.72	3.84	3.97	3.75	4.03	Yes
9^c^	8.1	AdV, *Mp*	0.1	4.3	2.61	4.57	3.92	1.85	5.38	Yes
10	9.9	Flu, MRSA	0.8	8.4	3.56	4.27	1.65	1.37	1.91	No
11	9.4	*Mp*	0.1	9.3	1.69	3.19	4.01	1.39	5.64	No
	6.3 (5.7, 7.6)^d^		0.8 (0.2, 2.8)^d^	6.4 (3.2, 8.6)^d^	2.61 (−3.20, −2.08)^d^	3.62 (−4.30, −3.15)^d^	2.02(1.70, 3.93)^d^	1.38 (1.25, 1.92)^d^	2.77(2.17, 4.37)^d^	

Abbreviations: AdV, adenovirus; BDR, bronchodilator response; CMV, cytomegalovirus; FEV1, forced expiratory volume in 1 second; Flu, influenza; FVC, forced vital capacity; hMPV, human metapneumovirus; HPIV‐3, human parainfluenza 3; HPIV‐4, human parainfluenza 4; *Mp*, *Mycoplasma pneumoniae*; MRSA, methicillin‐resistant staphylococcus aureus; PFT, pulmonary function test; R/E, rhinovirus/enterovirus; RSV, respiratory syncytial virus; RV, residual volume; TLC, total lung capacity.

^a^ Same patient identifiers as Table 1.

^b^ First PFT after infection.

^c^ Patient had PFT 1.5 years before inciting infection with a history of asthma. *Z*‐scores at that time: FEV1 −0.89, FVC −0.19, FEV1/FVC −1.33.

^d^ Median (interquartile range) for column.

**Table 3:** Pulmonary function outcomes in post‐infectious bronchiolitis obliterans.

PFT Parameter	Initial^a^	Final^a^	Change per year (95% CI)^b^	*p* value	ICC (95% CI)
FEV1	−3.83	−3.82	0.008 (−0.29, 0.045)	0.688	0.955 (0.894, 0.981)
FVC	−2.92	−1.76	0.185 (0.061, 0.309)	0.003	0.886 (0.752, 0.952)
FEV1/FVC	−2.61	−3.62	−0.143 (−0.239, −0.046)	0.004	0.934 (0.841, 0.974)

Abbreviations: CI, confidence interval; FEV1, forced expiratory volume in 1 second; FVC, forced vital capacity; ICC, intraclass correlation coefficient; PFT, pulmonary function test.

^a^ Median *z*‐score value of PFT parameter.

^b^ Average increase (positive) or decrease (negative) in PFT parameter *z*‐score for every year increase in time since infection.

**Figure 3:**
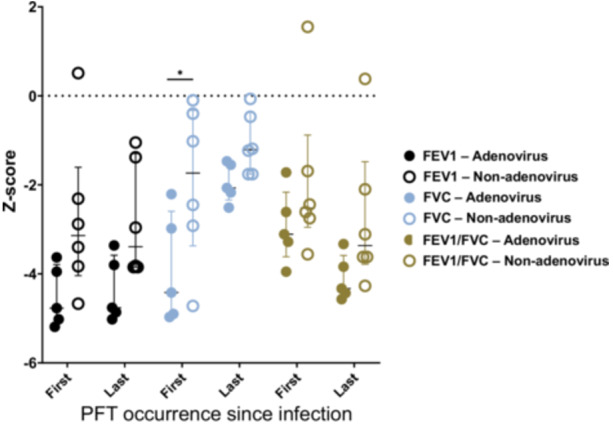
Adenovirus versus non‐adenovirus infected patient first and last spirometry parameters. FEV1 (black), FVC (blue), FEV1/FVC (gold). Solid circles represent adenovirus‐infected patients, open circles represent non‐adenovirus‐infected patients. Spirometry parameter *z*‐score versus spirometry occurrence since infection. Each bar represents the median with the interquartile range. *p = 0.02. [Color figure can be viewed at wileyonlinelibrary.com]

Next, spirometry was analyzed for those initially infected with adenovirus versus non‐adenovirus pathogens. At the time of initial spirometry, the median *z*‐score for FEV1 and FEV1/FVC did not differ in those initially infected with adenovirus (*n* = 5) versus non‐adenovirus (*n* = 6) (Figure 3). The initial FVC *z*‐score, however, was significantly lower in adenovirus‐infected patients (−4.42; IQR: −4.90, −2.98) versus the initial FVC *z*‐score of non‐adenovirus patients (−1.73; IQR: −2.80, −0.56; *p* = 0.02; Figure 3). At the time of final spirometry, the median *z*‐scores for FEV1, FVC, or FEV1/FVC did not differ between groups.

To further evaluate differences in the rate of change for spirometric measures between adenovirus and non‐adenovirus groups, mixed regression analyses were performed with respect to time. Patients with adenovirus infection had no significant change in FEV1 (*p* = 0.19) but demonstrated accelerated rates of FVC improvement (*p* < 0.001), leading to a decline in FEV1/FVC (*p* = 0.03) compared to non‐adenovirus patients (Supporting Information S2: E‐Figure 2A−C). Leave‐one‐out statistical analyses confirmed significance in FVC when each patient was individually excluded (Supporting Information S1: E‐Table 2). There were no significant interactions between time and steroid exposure, *M. pneumoniae* infection versus other pathogens, or age of infection for all pathogens combined (Supporting Information S2: E‐Figure 3). Height‐for‐age *z*‐score was also evaluated against time since infection, which showed no significant change over time when assessing all 11 patients as one cohort or when divided into patients infected with adenovirus versus those infected with other pathogens (Supporting Information S2: E‐Figure 4).

## 4 Discussion

The current study analyzed serial spirometry from children diagnosed with PIBO from a single center over 25 years. In children with PIBO, significant lung function impairment was present at time of first PFT occurrence with severely reduced FEV1, FVC, and FEV1/FVC *z*‐scores (Tables 2 and 3). Across 6 years of serial spirometry, FEV1 remained persistently low with a z‐score below −3.8 at the time of the final spirometry event. In contrast, there was significant growth in FVC, rising from a *z*‐score of −2.92 to −1.76, resulting in a decline in the FEV1/FVC ratio. This data demonstrates that dysanaptic lung growth develops in PIBO due to stagnant airway, but with sustained distal parenchymal growth. These trends in dysanaptic lung growth were also associated with the inciting pathogen, specifically initial adenovirus infection in children affected by PIBO.

Lung dysanapsis is defined as an imbalance of distal lung parenchymal growth compared to proximal airway growth [27]. Consistent with prior PIBO cohorts [10, 14, 15, 16], the current study shows significant dysanaptic lung growth with impaired FEV1 growth, but with continued FVC growth, in children affected by PIBO. In contrast to prior publications, this study demonstrated that the FVC growth is non‐linear, with notable FVC growth within the first 2 years after infection (Figure 2). Non‐linear FVC improvement in the first 2 years after infection is different from previously published case series on PIBO spirometry [10, 14, 16]. The age at the time of infection could impact the spirometry outcomes that are obtained. Our cohort has an older age of infection with a median of 3 years, while other cohorts have a younger age of infection [10, 14, 16, 28]. The older age allows for a sooner pulmonary function test, which would identify these non‐linear changes in contrast to patients infected < 2 years of age who are unable to perform spirometry for multiple years following infection. It is also important to recognize the potential optimization for pores or Kohn, channels of Martin, and canals of Lambert in the early disease course, potentially allowing for easier movement of air out of distended alveoli and improving air movement [29]. Additionally, different strains of respiratory tract infections must be considered as a source of differences in pulmonary function trajectory. The United States has little data in PIBO literature, and viruses of different strains may impact children in our cohort, as opposed to other countries. Viral serotyping is infrequently performed clinically but could help to phenotype patients with more virulent strains, such as adenovirus 3, 5, 7, and 21 reported in prior PIBO cohorts [30]. In parallel, unidentified differences in genetic susceptibility are still to be ascertained. There is no known genetic link to the development of PIBO, but genetic predisposition has been suggested [31, 32, 33].

The current study also identified critical differences in lung growth trajectories when comparing inciting infection. FVC was significantly lower in adenovirus‐infected patients versus non‐adenovirus patients with a *z*‐score 2.69 points lower at initial spirometry (*p* = 0.02; Figure 3). However, this difference in FVC *z*‐scores between adenovirus and non‐adenovirus patients resolved by the time of final spirometry to a difference of just 0.8 points lower (*p* > 0.05). These results suggest that adenovirus results in more severe lung impairment initially, but with accelerated rates of FVC recovery years after infection compared to non‐adenovirus counterparts. In contrast, FEV1 *z*‐score was −4.79 for patients infected with adenovirus. This is 1.5 *z*‐score points lower than those with PIBO from other infections, albeit not statistically significant. Following 6 years of PFT monitoring post‐infection, this difference in FEV1 remained unchanged, highlighting persistent and severe airway obstruction with failed recovery. Of note, the two patients infected with adenovirus older than 5 years old were also co‐infected with *M. pneumoniae* (Table 1). In both cases, it was unclear if one pathogen was more influential in the disease process, as both are strongly implicated in PIBO development. The pathophysiology of this difference in pathogen has not been elucidated; however, independent of the underlying mechanism, these results provide improved resolution into the typical lung function trajectories of patients with adenovirus‐induced PIBO.

Continued controversy exists over whether PIBO causes a fixed or partially reversible obstruction. Recent evidence suggests phenotypic differences in some PIBO patients who demonstrate bronchodilator responsiveness [10, 11, 12, 13]. Results from this study also support bronchodilator responsiveness with four out of nine patients tested showing a positive bronchodilator response. Of note, one patient with asthma had normal spirometry prior to infection and remained bronchodilator responsive after PIBO diagnosis (Table 1). Limited studies suggest that asthma is a potential independent risk factor for developing PIBO [34, 35]. Equally important, these results emphasize the continued need to evaluate whether a PIBO patient transitions from variable to fixed obstructive phenotype. All patients who were responsive to bronchodilators received either ICS or combined ICS+long‐acting beta agonist therapy. Only one of the four patients with bronchodilator responsiveness received pulse dose or long‐term oral steroids. Larger studies are required to provide sufficient power to determine if a certain sub‐cohort of PIBO patients are bronchodilator responsive, which may benefit more from systemic steroid treatment.

Limitations of this study include a small sample size and a single‐center focus. When addressing a rare disease, these factors limit the generalizability of the results. There is potential for overfitting when using restricted splines in mixed effects modeling, as was used in this study. This was mitigated by using a standard restricted cubic spline approach that avoids arbitrary placement by ensuring adequate data density [36]. Misclassification and ascertainment bias are possible based on the clinical diagnostic nature that PIBO can require, as there is no diagnostic test outside of biopsy. In exploratory analyses, three patients had unknown infections. These were treated as non‐adenovirus infections, and one had negative adenovirus titers. It is not clear what this classification may have done to alter results. Four patients did not have PFT data available. Three of these were too young to perform spirometry. One patient was deemed too ill to perform PFTs by the treating physician. Given the small sample size, the impact of this patient's lung function trajectory could introduce an unrecognized bias toward favorable FVC. Selection bias is important to consider, particularly given the small sample size. Attempts were made to avoid selection bias by establishing 5 years old at the time of infection as a division point for exploratory subgroup analysis, since this is an age where spirometry can reasonably be performed. In our study population, the adenovirus group was defined based on the presence of adenovirus at the time of the sentinel respiratory infection, regardless of whether it occurred as a single infection or as part of a mixed viral infection. Because of the limited sample size, we did not perform a separate analysis of single adenovirus infection versus multi‐pathogen adenovirus co‐infection. Overall, however, the data from this study align with results of other studies that have been published in the literature with long‐term PFT surveillance in patients with PIBO [10, 14, 15, 16]. Additionally, given the relatively little data that has been published, this study strengthens the available research and adds more data from the United States, an area that has been underrepresented in PIBO literature. Larger studies are necessary to further understand the pulmonary function changes in patients living with PIBO.

In conclusion, spirometry is an effective way to monitor patients with PIBO. The present cohort demonstrated dysanaptic lung growth with stagnant gains in FEV1 but improved rates of change in FVC, most notable in the first 2 years after infection and following adenovirus infection. In older children affected by PIBO, FEV1 is a sensitive marker for monitoring disease progression and therapeutic response in PIBO. Larger studies are needed to further characterize these dysanaptic changes, particularly in the immediate years after infection, as this is an important window for disease modification and intervention.

## Author Contributions

**James M. Clegg:** conceptualization (equal), investigation (lead), methodology (equal), writing – original draft (lead), writing – review and editing (equal). **Ferdinand Cacho:** formal analysis (lead), writing – review and editing (equal). **Matthew D. McGraw:** conceptualization (supporting), formal analysis (supporting), supervision (supporting), writing – review and editing (equal). **Michael G. O'Connor:** conceptualization (equal), methodology (equal), supervision (lead), writing – review and editing (equal).

## Ethics Statement

The internal review board at Vanderbilt University Medical Center reviewed the study protocol and granted exempt status because it posed minimal risk to participants. It was approved as IRB #242149. It was held to the standards of the US Federal Policy for the Protection of Human Subjects.

## Conflicts of Interest

The authors declare no conflicts of interest.

## Supporting information

Supporting File 1

Supporting File 2

## Data Availability

The data that support the findings of this study are available on request from the corresponding author. The data are not publicly available due to privacy or ethical restrictions.
