# Supplementary material for: Non‐Linear Dysanaptic Lung Growth in Patients With Post‐Infectious Bronchiolitis Obliterans
Source: Pediatr Pulmonol. 2026 Jul 3;61(7):e71716. doi: 10.1002/ppul.71716 (PMC13330942; doi:10.1002/ppul.71716)
Supplement: Supplementary file 1 — Supporting File 1 [file PPUL-61-0-s002.docx]

E-figure 1. Relationship of total lung capacity (TLC) and residual volume (RV) with respect to time since infection. Generalized mixed-effects regression models were used to evaluate changes in pulmonary function over time, with restricted maximum likelihood estimation (REML) applied for parameter estimation. Population trendline (navy blue line), 95% confidence interval (shaded area), and individual pulmonary function outcomes (open circles with dashed lines) are represented. A: TLC z-score vs time since infection. B: RV z-score vs time since infection. C: RV/TLC z-score vs time since infection. (*p*>0.05)

E-figure 2. A-C: The interaction effect of adenovirus infection and time since infection on spirometry parameters for patients with PIBO. Adenovirus trend (solid black line) and 95% CI (tan shaded area) and all other pathogens (dashed black line) and 95% CI (blue shaded area). D-F: The interaction effect of age and time since infection on lung function parameters for patients with adenovirus-associated PIBO. Trend of patients >5 yrs (solid black line) and 95% CI (tan shaded area) and patients <5 yrs (dashed black line) and 95% CI (blue shaded area) at time of infection. A/D: FEV1 z-score vs time since infection. B/E: FVC z-score vs time since infection. C/F: FEV1/FVC ratio z-score vs time since infection. (***p<*0.01, ****p<*0.001)

E-figure 3. Exploratory analysis comparing various parameters vs time since infection. A-C: The interaction effect of steroid exposure, defined as receiving either prolonged oral steroids or intravenous pulse-dose steroids, and time since infection on lung function parameters for patients with PIBO. Steroid exposure trend (solid black line) and 95% confidence interval (CI) (tan shaded area) and no steroid exposure trend (dashed black line) and 95% CI (blue shaded area). D-F: The interaction effect of *M. pneumoniae* infection and time since infection on lung function parameters for patients with PIBO. *Mycoplasma* trend (solid black line) and 95% CI (tan shaded area) and all other pathogens (dashed black line) and 95% CI (blue shaded area). G-I: The interaction effect of age at infection and time since infection on lung function parameters for patients with PIBO. Age >5 years at infection trend (solid black line) and 95% CI (tan shaded area) and age <5 years at infection (dashed black line) and 95% CI (blue shaded area). A/D/G: FEV1 z-score vs time since infection. B/E/H: FVC z-score vs time since infection. C/F/I: FEV1/FVC ratio z-score vs time since infection. No interactions A-I were statistically significant.

E-figure 4. A: Relationship of height and time since infection. Generalized mixed-effects regression models were used to evaluate changes in pulmonary function over time, with restricted maximum likelihood estimation (REML) applied for parameter estimation. Population trendline (navy blue line), 95% confidence interval (shaded area), and individual pulmonary function outcomes (open circles with dashed lines) are represented. B: The interaction effect of adenovirus infection and time since infection with respect to height for patients with PIBO. Adenovirus trend (solid black line) and 95% CI (tan shaded area) and all other pathogens (dashed black line) and 95% CI (blue shaded area). Neither A nor B were statistically significant.

| E-table 1. Post-hospital interventions | | | | | | | | | |  |
| --- | --- | --- | --- | --- | --- | --- | --- | --- | --- | --- |
| Patient | Home O2 | Long-term Oral or Pulse steroids | Azithromycin | Airway Clearance | IVIG | ICS | Other | PFT |  |  |
| 1 | Yes | Yes | - | - | - | ICS/LABA | - | Yes |  |  |
|  |  |  |  |  |  |  |  |  |  |  |
| 2 | - | - | Yes | - | - | ICS | - | Yes |  |  |
|  |  |  |  |  |  |  |  |  |  |  |
| 3 | - | - | - | Yes | - | - | - | Yes |  |  |
|  |  |  |  |  |  |  |  |  |  |  |
| 4 | - | - | - | - | - | ICS | - | Yes |  |  |
|  |  |  |  |  |  |  |  |  |  |  |
| 5 | Yes | Yes | Yes | Yes | - | ICS/LABA | - | Yes |  |  |
|  |  |  |  |  |  |  |  |  |  |  |
| 6 | - | - | - | - | - | ICS/LABA | - | Yes |  |  |
|  |  |  |  |  |  |  |  |  |  |  |
| 7 | - | Yes | Yes | Yes | - | ICS/LABA | - | Yes |  |  |
|  |  |  |  |  |  |  |  |  |  |  |
| 8 | - | - | Yes | Yes | - | ICS/LABA | - | Yes |  |  |
|  |  |  |  |  |  |  |  |  |  |  |
| 9 | Yes | Yes | Yes | Yes | Yes | ICS | etanercept, tiotropium | Yes |  |  |
|  |  |  |  |  |  |  |  |  |  |  |
| 10 | - | Yes | Yes | Yes | - | ICS/LABA | montelukast | Yes |  |  |
|  |  |  |  |  |  |  |  |  |  |  |
| 11 | Yes | Yes | Yes | - | Yes | ICS/LABA | trimethoprim/ sulfamethoxazole ppx | Yes |  |  |
|  |  |  |  |  |  |  |  |  |  |  |
| 12 | - | - | - | Yes | - | ICS | - | No |  |  |
|  |  |  |  |  |  |  |  |  |  |  |
| 13 | Yes | Yes | Yes | Yes | - | ICS | trimethoprim/ sulfamethoxazole ppx | No |  |  |
|  |  |  |  |  |  |  |  |  |  |  |
| 14 | - | - | Yes | Yes | - | ICS | montelukast | No |  |  |
|  |  |  |  |  |  |  |  |  |  |  |
| 15 | - | Yes | - | - | - | - | - | No |  |  |
| Abbreviations: ICS – inhaled corticosteroid, IVIG – intravenous immunoglobulin, LABA – long-acting beta agonist, ppx - prophylaxis | | | | | | | | |  |  |
|  | | | | | | | | | | |

E-table 2. Leave-one-out sensitivity analysis for adenovirus x time since infection interaction

|  | Omitted participant with Adenovirus infection | Interaction estimate (β coefficient) | 95% confidence interval | p-value |
| --- | --- | --- | --- | --- |
| FEV1 | Subject A | 0.059 | -0.019, 0.138 | 0.140 |
|  | Subject B | 0.052 | -0.028, 0.133 | 0.203 |
|  | Subject C | 0.045 | -0.043, 0.134 | 0.317 |
|  | Subject D | 0.036 | -0.063, 0.136 | 0.475 |
|  | Subject E | 0.064 | -0.027, 0.156 | 0.169 |
| FVC | Subject A | 0.344 | 0.202, 0.487 | <0.001 |
|  | Subject B | 0.352 | 0.207, 0.497 | <0.001 |
|  | Subject C | 0.314 | 0.152, 0.476 | <0.001 |
|  | Subject D | 0.239 | 0.095, 0.384 | 0.001 |
|  | Subject E | 0.437 | 0.321, 0.553 | <0.001 |
| FEV1/FVC | Subject A | -0.171 | -0.340, -0.001 | 0.047 |
|  | Subject B | -0.206 | -0.379, -0.034 | 0.019 |
|  | Subject C | -0.164 | -0.353, 0.024 | 0.088 |
|  | Subject D | -0.112 | -0.279, 0.054 | 0.186 |
|  | Subject E | -0.268 | -0.416, -0.121 | <0.001 |
