# Supplementary material for: Non‐Linear Dysanaptic Lung Growth in Patients With Post‐Infectious Bronchiolitis Obliterans
Source: Pediatr Pulmonol. 2026 Jul 3;61(7):e71716. doi: 10.1002/ppul.71716 (PMC13330942; doi:10.1002/ppul.71716)
Supplement: Supplementary file 2 — Supporting File 2 [file PPUL-61-0-s001.pptx]

## Slide 1
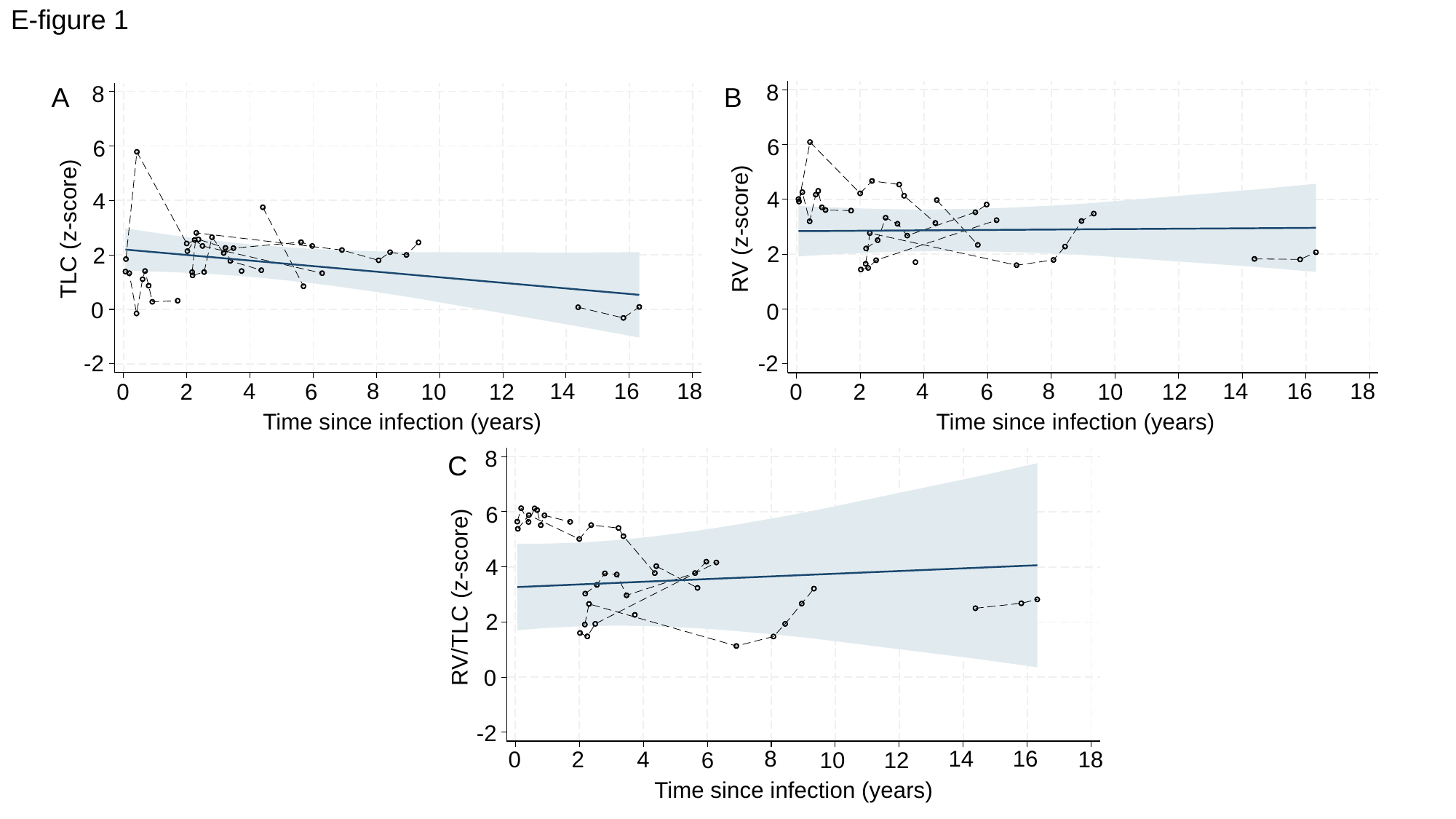

E-figure 1
8
8
B
A
6
6
4
4
TLC (z-score)
RV (z-score)
2
2
0
0
-2
-2
8
14
16
8
14
16
4
4
18
18
0
2
0
2
6
10
12
6
10
12
Time since infection (years)
Time since infection (years)
8
C
6
4
RV/TLC (z-score)
2
0
-2
8
14
16
4
18
0
2
6
10
12
Time since infection (years)

## Slide 2
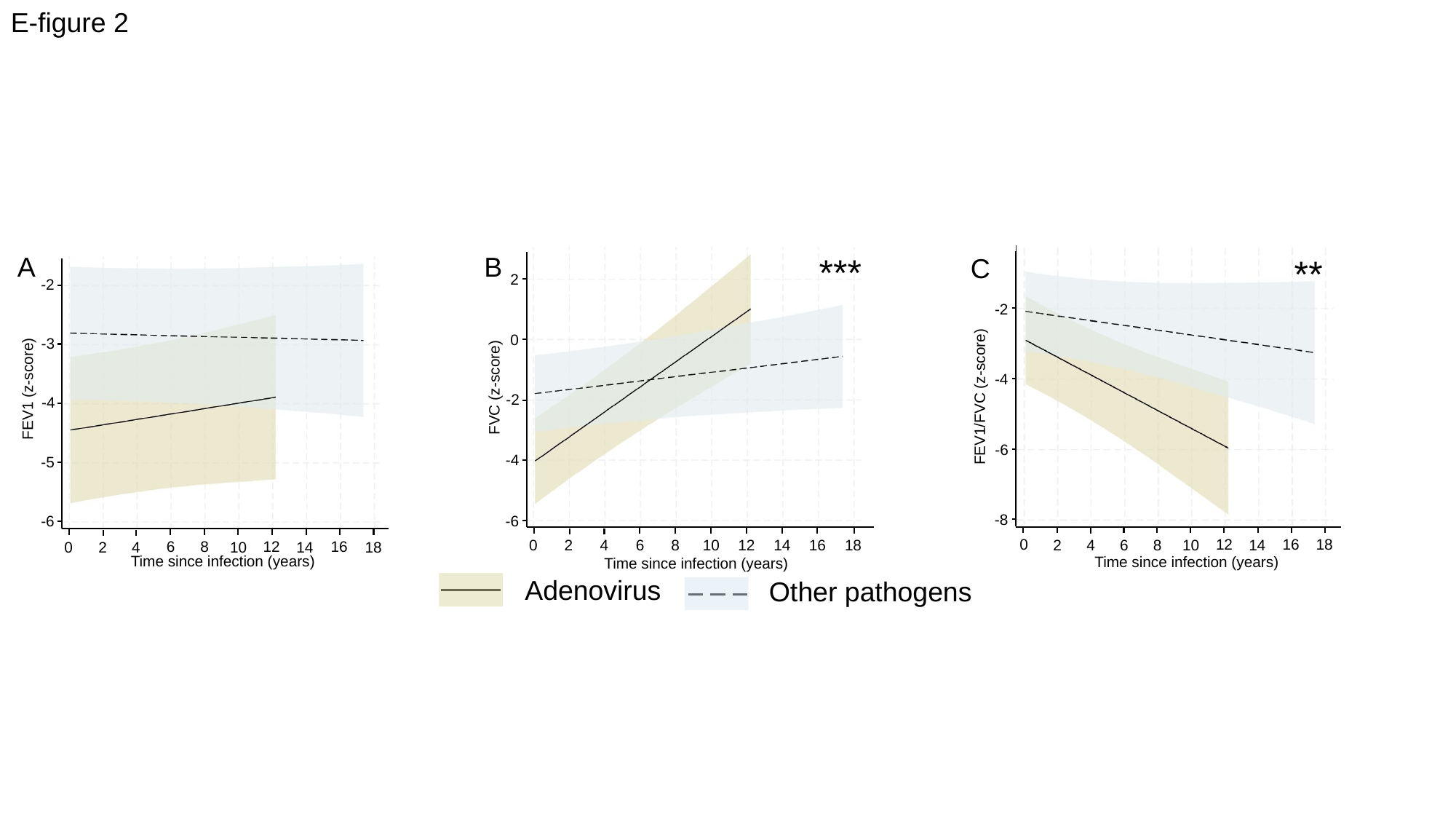

E-figure 2
***
A
B
C
**
2
-2
-2
0
-3
-4
FVC (z-score)
FEV1 (z-score)
FEV1/FVC (z-score)
-2
-4
-6
-4
-5
-8
-6
-6
18
12
0
16
10
2
0
4
12
16
6
10
14
18
2
6
8
14
4
8
12
16
8
6
10
14
18
4
2
0
Time since infection (years)
Time since infection (years)
Time since infection (years)
Adenovirus
Other pathogens

## Slide 3
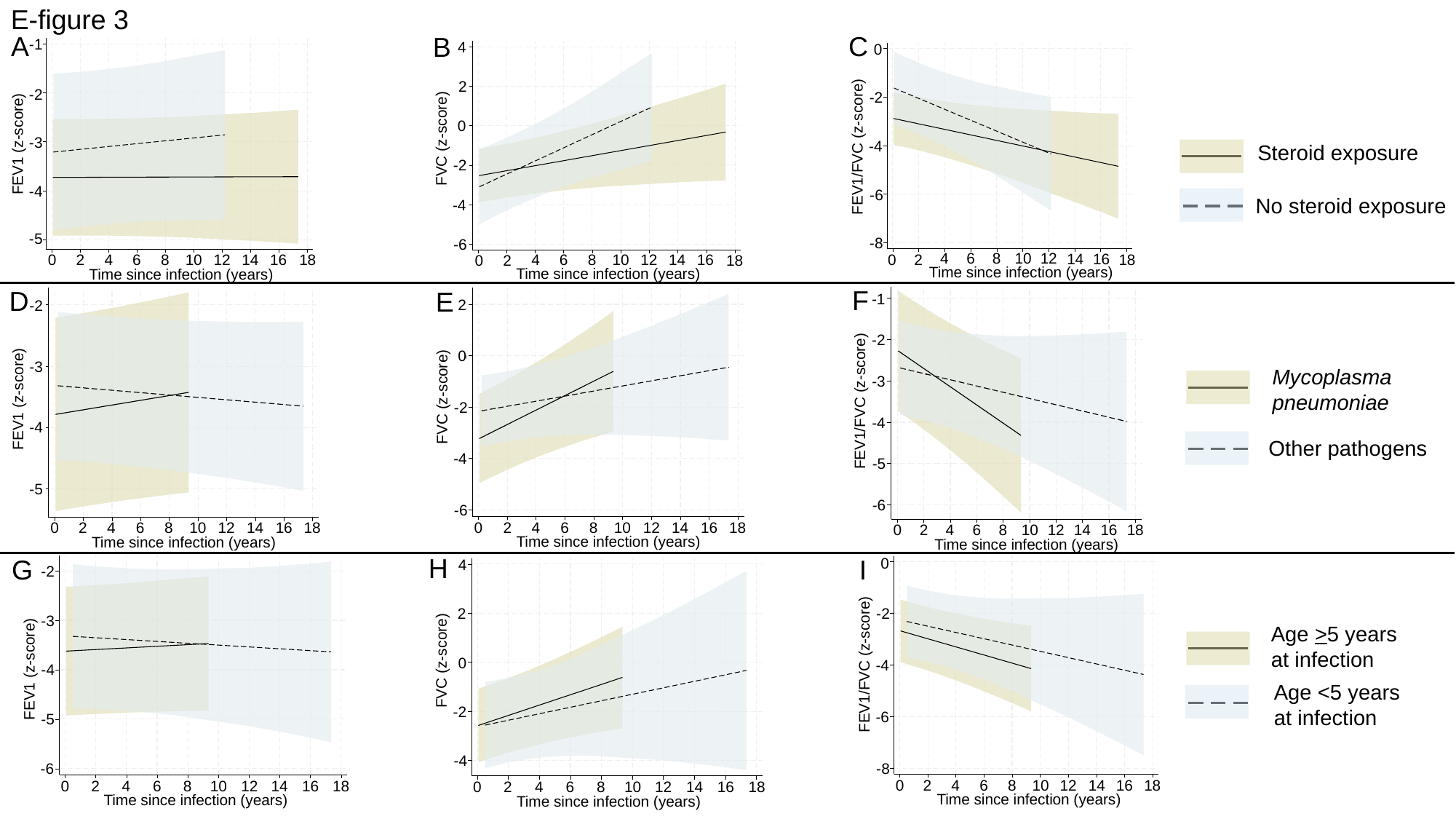

E-figure 3
C
0
-2
FEV1/FVC (z-score)
-4
-6
-8
4
6
8
10
12
14
16
0
2
18
Time since infection (years)
A
-1
-2
FEV1 (z-score)
-3
-4
-5
0
2
4
6
8
10
12
14
16
18
Time since infection (years)
B
4
2
0
FVC (z-score)
-2
-4
-6
4
6
8
10
12
14
16
0
2
18
Time since infection (years)
Steroid exposure
No steroid exposure
F
-1
-2
-3
FEV1/FVC (z-score)
-4
-5
-6
0
2
4
6
8
10
12
14
16
18
Time since infection (years)
D
-2
-3
FEV1 (z-score)
-4
-5
0
2
4
6
8
10
12
14
16
18
Time since infection (years)
E
2
0
FVC (z-score)
-2
-4
-6
0
2
4
6
8
10
12
14
16
18
Time since infection (years)
Mycoplasma
pneumoniae
Other pathogens
H
G
-2
-3
FEV1 (z-score)
-4
-5
-6
0
2
4
6
8
10
12
14
16
18
Time since infection (years)
I
0
4
-2
2
Age >5 years
at infection
FVC (z-score)
FEV1/FVC (z-score)
0
-4
Age <5 years
at infection
-2
-6
-4
-8
0
2
4
6
8
10
12
14
16
18
0
2
4
6
8
10
12
14
16
18
Time since infection (years)
Time since infection (years)

## Slide 4
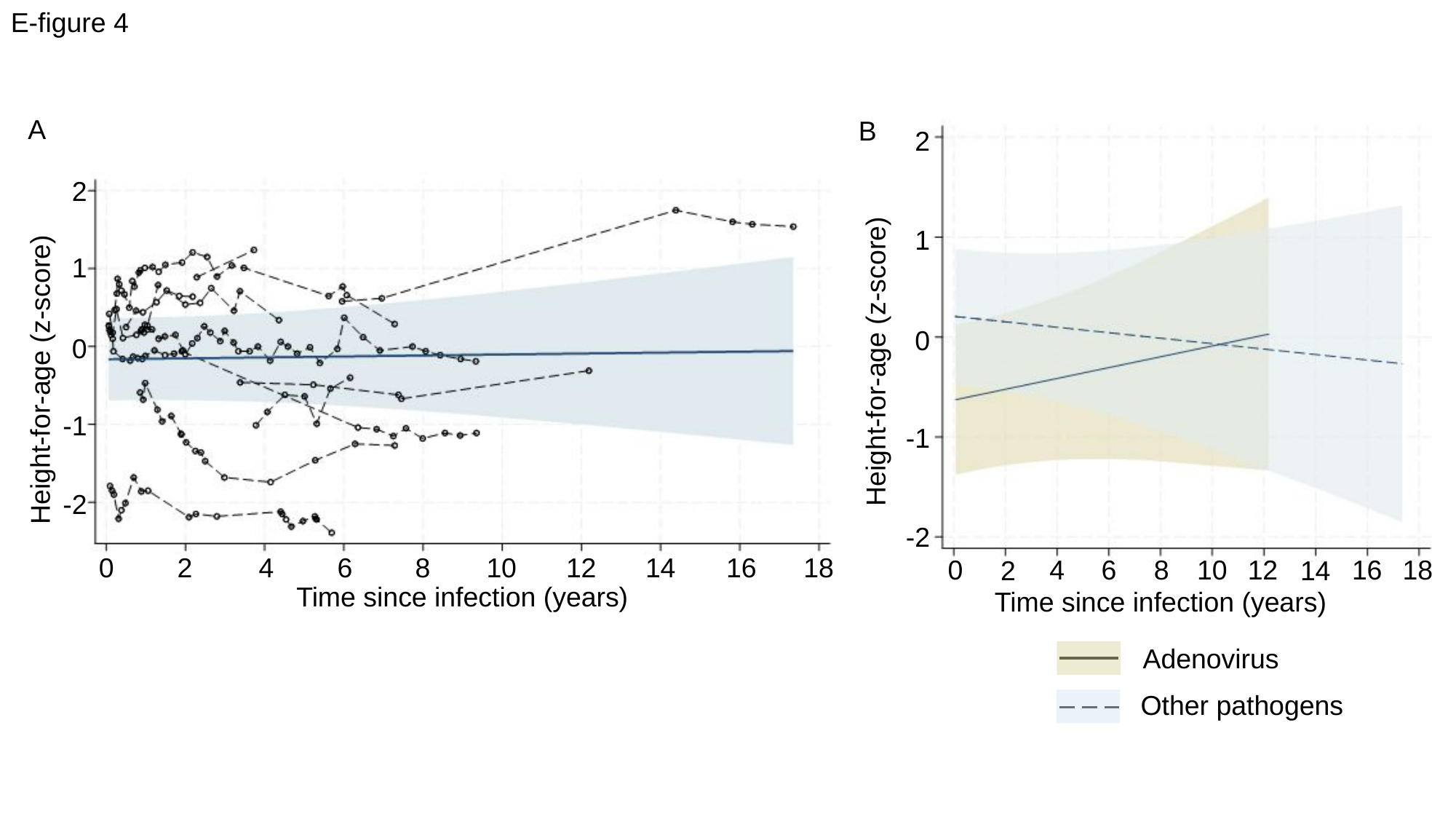

E-figure 4
A
B
2
2
1
1
0
0
Height-for-age (z-score)
Height-for-age (z-score)
-1
-1
-2
-2
0
4
8
10
6
12
16
18
2
14
0
4
8
10
6
12
16
18
2
14
Time since infection (years)
Time since infection (years)
Adenovirus
Other pathogens
